# Metabolic fate of chokeberry (*Aronia melanocarpa*) phenolics in different food matrices

**DOI:** 10.1016/j.crfs.2024.100967

**Published:** 2024-12-24

**Authors:** Magdalena Köpsel, Gulay Ozkan, Tuba Esatbeyoglu

**Affiliations:** a Department of Molecular Food Chemistry and Food Development, Institute of Food and One Health, Gottfried Wilhelm Leibniz University Hannover, Am Kleinen Felde 30, 30167 Hannover, Germany; b Department of Food Engineering, Faculty of Chemical and Metallurgical Engineering, Istanbul Technical University, 34469, Maslak, Istanbul, Türkiye

**Keywords:** Antioxidant activity, Aronia, Berry, Bioaccessibility, Bioavailability, Bioactive, Cell culture, *In vitro* digestion, Milk, Polyphenol

## Abstract

Chokeberry (*Aronia melanocarpa*) has been traditionally used as a folk remedy due to its health-promoting effects. The aim of this study was to investigate the effects of chokeberry polyphenols combined with the matrices of milk and milk alternatives on the permeability of the intestinal barrier. Based on this, *in vitro* availability of chokeberry polyphenols was tested by gastrointestinal model combined with a co-culture of human colon adenocarcinoma cells (Caco-2) and human colon cancer cells (HT29-MTX). Additionally, the antioxidant capacity of the samples was analyzed by DPPH (2,2-diphenyl-1-picrylhydrazyl) and ABTS (2,2′-azino-bis(3-ethylbenzothiazoline-6-sulfonic acid) assays. According to the results, both chokeberry juice and chokeberry juice in combination with milk showed a higher recovery of DPPH radical scavenging ability after intestinal digestion. Moreover, a significant difference in the transport of Lucifer Yellow through the intestinal membrane was observed when compared to the control. Therefore, fat- and protein-rich food matrices could represent a potential to increase the bioavailability of phenolic compounds while reducing intestinal barrier injury.

## Highlights

• Higher total phenol content in aronia juice with lactose-free milk or oat drink.
• Higher antioxidant potential in aronia juice after intestinal digestion.
• No effect of lactose and fat content of milk on the intestinal permeability.
• Reduction in the permeability coefficient with aronia juice and low-fat milk.

## Graphical abstract

**Figure:**
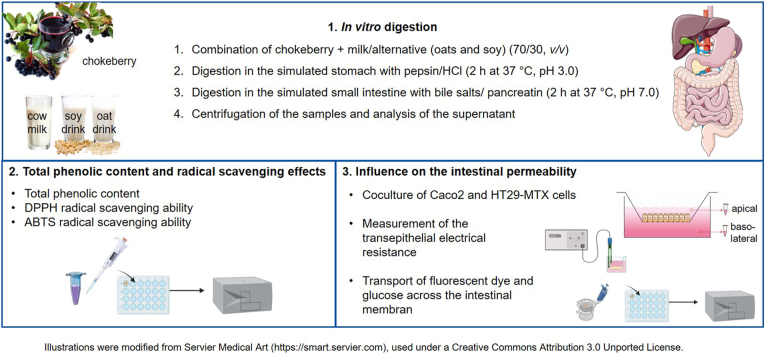

## 1 Introduction

Fruit and vegetables contain several health-promoting ingredients, including fiber, vitamins, minerals and phytochemicals, such as polyphenols. These bioactive compounds found in fruit and vegetables can help protect human health against chronic, degenerative diseases (Dragovic-Uzelac et al., 2007; Treutter, 2006). In addition, epidemiological studies have long indicated that foods rich in polyphenols can reduce the risk of cardiovascular disease and cancer (Lotito and Frei, 2006). Consequently, interest in the potential cancer-preventive and therapeutic properties of plant polyphenols from food has increased significantly in recent years (Fresco et al., 2006). Berries such as chokeberry (*Aronia melanocarpa*), elderberry (*Sambucus nigra*) and cranberry (*Vaccinium macrocarpon*) contain high amounts of valuable bioactive substances such as flavonoids and phenolic acids (Gośliński et al., 2021; Manganaris et al., 2014; Olas, 2018). Their antioxidant effect is already well described in the scientific literature, with antibacterial, anti-inflammatory, antidiabetic, anticancer, cardioprotective and gastroprotective properties also being attributed to these compounds (Nowak et al., 2022). However, the bioaccessibility of biocomponents from foods is also an essential parameter for assessing the relationship between foods and their health benefits (Dima et al., 2020). Bioaccessibility, bioavailability and transport through the intestinal epithelium into the blood, as well as microbiological digestion in the large intestine, are important factors that have a strong impact on the bioactivity of polyphenols. To date, there are a few studies in which fruits or fruit juices have been subjected to *in vitro* gastrointestinal digestion (Bermúdez-Soto et al., 2007). Some authors investigated the behavior of food ingredients in individual compartments of the gastrointestinal tract to analyze the release of biocomponents, their solubility and interaction, as well as their digestibility and bioaccessibility of biocomponents under simulated physiological conditions of the small intestine (Winuprasith et al., 2018). Other authors evaluated the bioaccessibility of food ingredients using multistage gastrointestinal models in which food is sequentially passed through all simulated fluids specific to the upper gastrointestinal tract compartments (Qin et al., 2017).

Permeability of the intestinal barrier has been shown to contribute to systemic inflammation observed in obesity, inflammatory bowel disease and other chronic diseases (Calder et al., 2011; Moreno-Navarrete et al., 2012). Increased permeability of the intestinal barrier also promotes the translocation of endotoxins and other pro-inflammatory components into the lumen, thereby perpetuating chronic inflammation (Cani et al., 2007). These inflammatory signals and other endogenous cytokines can therefore directly influence the intestinal barrier by reducing the localization and expression of tight junction proteins (Cui et al., 2010). Dietary components such as probiotics, polyphenols, lipids and proteins could improve gut barrier function by inhibiting inflammation or directly influencing barrier function (Carrasco-Pozo et al., 2013; Anderson et al., 2010). In most studies investigating intestinal permeability, the Caco-2 cell model was used. The Caco-2 cells are easily differentiated and form a layer of enterocyte cells with a morphology very similar to the epithelial layer of the intestine. Studies have shown that the monolayer of Caco-2 cells has digestive enzymes such as peptidases, but also active membrane transporters such as glycoprotein, sugar and vitamin transporters, etc. (Costa and Ahluwalia, 2019). For a more realistic mimicry of the intestinal epithelial layer, some researchers have developed dual co-culture models with HT29-MTX cells that ensure mucus production and goblet cell growth in the Caco-2 cell monolayer (Dima et al., 2020; Antunes et al., 2013).

However, many fruit compounds have low oral bioaccessibility due to their low stability in the gastrointestinal fluid and consequently poor absorption through the epithelial layer of the gut (Rafiee et al., 2019). A fat-rich matrix, as is the case with milk, could also improve the bioaccessibility of polyphenols. Fruit drinks are often commercially fortified with milk, vitamins and/or minerals to improve their nutritional value and provide so-called bioactive components. Dairy products are rich in bioactive compounds and probiotic microorganisms that can influence the immune system and metabolism and promote human health beyond nutrition (Meydani and Ha, 2000; Adolfsson et al., 2004; Astrup, 2014). Dietary components such as probiotics, polyphenols, lipids and proteins could improve the function of the intestinal barrier by inhibiting inflammation or by directly influencing the barrier function (Carrasco-Pozo et al., 2013; Anderson et al., 2010). Epidemiological studies have shown that the consumption of dairy products has the potential to improve intestinal health, prevent constipation and diarrhoea and reduce the risk of colon cancer (Adolfsson et al., 2004; Astrup, 2014).

In recent years, some studies have emphasized how flavonoids can interact with proteins, which could lead to various structural changes (Cao and Xiong, 2017; Pal et al., 2012; Shen et al., 2014). Among proteins, milk proteins have been investigated for interaction with flavonoids, mainly because of the high binding affinity of some dietary flavonoids for milk proteins, both in pure compounds, as well as in natural extracts (Han et al., 2022; Yildirim-Elikoglu and Erdem, 2018). As a result of protein/flavonoid interaction, the digestibility of proteins could also be affected. In products containing proteins (e.g. dairy products), the addition of flavonoids as additives in fortified foods has shown a reduction in protein digestibility with improved stability of the flavonoid/protein complex (Swieca et al., 2014).

Therefore, previous studies have attempted to improve the bioaccessibility of phenolic compounds by adding different food matrices to fruit juices. For example, Ozkan et al. (2022) investigated the fate of polyphenols in cranberry juice in combination with bovine milk and almond milk to evaluate the influence of the milk matrix on bioaccessibility and bioavailability. According to the studies, the addition of milk appears to promote the transepithelial transport of chlorogenic acid (Ozkan et al., 2022). It is believed that a number of findings from the studies investigating the effects of the milk matrix are likely to be related to various protein-polyphenol interactions. In this regard, the milk matrix may protect phenolics from degradation in the gut and promote the release and availability of phenolic compounds throughout the gastrointestinal tract (Ozkan et al., 2022; Rodríguez-Roque et al., 2014). In addition, beverage mixes enriched with milk alternatives, such as soy and oats, can be an alternative for people who are vegetarian or have a lactose intolerance (Kundu et al., 2018).

In view of the above, the aim of the present study was to evaluate the stability, bioaccessibility and bioavailability of the phenolic compounds in chokeberry-based juices using simulated gastrointestinal digestion, coupled with the co-cultured Caco2 and HT29-MTX cell model. In the current investigation, we have examined (i) the phenolic content and antioxidant capacity of chokeberry juice-based beverages, (ii) changes in the phenolics and antioxidant capacity of the samples during *in vitro* gastrointestinal digestion (bioaccessibility), (iii) effects of various food matrices on the transport dynamics of chokeberry phenolics (bioavailability).

## 2 Materials and methods

### 2.1 Chemicals

Double deionized water was obtained from Elga Labwater Purelab Flex 3 (Veolia, Celle, Germany). 2,2′-Azino-bis-(3-ethylbenzothiazoline-6-sulfonic acid) (ABTS, 98%), ammonium carbonate, bile, calcium chloride dihydrate, carbazole, 2,2-diphenyl-1-picrylhydrazyl (DPPH), galacturonic acid monohydrate, pepsin (541 U/mg, EC 3.4.23.1), pancreatin (8x USP, EC 232.468.9), potassium sodium tartrate tetrahydrate, copper (II) sulfate-5-hydrate, sodium hydrogen carbonate, sodium fluorescein and hydrochloric acid (37%) were obtained from Sigma-Aldrich (Steinheim, Germany) and Merck KGaA (Darmstadt, Germany). Acetonitrile (HPLC grade) and acetic acid (HPLC grade) were purchased from VWR International (Leuven, Belgium). Folin-Ciocalteu reagent and dimethyl sulfoxide, p.a. were purchased from Merck KGaA (Darmstadt, Germany). Ethanol (denatured with methyl ethyl ketone, 99%) was purchased from Walter CMP (Kiel, Germany). Gallic acid monohydrate and 6-hydroxy-2,5,7,8-tetramethylchroman-2-carboxylic acid (Trolox, ≥98% purity) were purchased from Fluka (Buchs, Switzerland). Formic acid (HPLC grade), methanol (HPLC grade), ethyl acetate (≥99% p.a.) and PBS were purchased from Th. Geyer (Höxter, Germany). Capric acid (C10), acetic acid (conc.), potassium chloride (≥99% p.a.), potassium hydrogen phosphate (≥99% p.a.), sodium chloride (≥99% p.a.), sodium hydroxide (≥98% p.a.), Triton X-100 and trypan blue solution (0.4%) were purchased from Carl Roth (Karlsruhe, Germany). Dulbecco's Modified Eagle Medium (DMEM), Fetal Calf Serum Standard (FCS), Hanks' Balanced Salt Solution (HBSS, without phenol red, with Ca^2+^, Mg^2+^ and 0.35 g/L NaHCO_3_), MEM Non Essential Amino Acid Solution (NEAAS), penicillin-streptomycin and trypsin/EDTA were purchased from PAN Biotech (Aidenbach, Germany). Glucose- Colorimetric assay kit was purchased from Elabscience (Texas, USA).

### 2.2 Preparation of chokeberry juice-based beverages

Chokeberry juice was kindly provided by the company of Aronia Original (Dresden, Germany). The bovine whole milk, bovine milk, lactose-free bovine milk, soy drink and oat drink were purchased at a local supermarket (Hanover, Germany). The nutritional composition of the bovine whole milk consisted of 3.6% (w/v) fat, 3.3% (w/v) protein and 5.0% (w/v) carbohydrate, whereas 1.6% (w/v) fat, 3.4% (w/v) protein and 5.1% (w/v) carbohydrate were included in bovine milk. The nutritional composition of lactose-free bovine milk comprised 3.6% (w/v) fat, 3.3% (w/v) protein and 5.0% (w/v) carbohydrate. The nutritional composition of the oat drink consisted of 1.4% (w/v) fat, 0.6% (w/v) protein and 6.2% (w/v) carbohydrate, while 1.9% (w/v) fat, 3.2% (w/v) protein and 1.8% (w/v) carbohydrate were included in soy drink (data provided by manufacturers).

For the production of fruit juice mixtures, 75% of the chokeberry juice and 25% of bovine milk, soy drink or oat drink were mixed. The formulation of the drink was selected on the basis of previous studies (Ozkan et al., 2022; Rodríguez-Roque et al., 2014). All digestion samples and biochemical determinations were performed in three replicates and used immediately in simulated gastrointestinal digestion studies.

### *2.3* *In vitro* simulated gastrointestinal digestion

The *in vitro* gastrointestinal digestion method was performed based on Brodkorb et al. (2019) with some modifications. This method involves three sequential steps. Firstly, for the oral digestion phase, 5 mL of each chokeberry-based beverage sample was mixed with 4 mL simulated salivary fluid, 25 μL CaCl_2_ and 975 μL double deionized water and incubated in a water bath (GFL 1092, Burgwedel, Germany) at 37 °C and 150 rpm for 2 min. Amylase is required for the digestion of starchy or solid foods. Therefore, it was excluded from the following description of the experiment, as the sample solutions did not contain starch. To simulate gastric digestion, the pH of the samples was adjusted to 3.0 with 1 M HCl after addition of 6 mL simulated gastric fluid and 556 μL double deionized water and then porcine pepsin was added at a final concentration of 1.3 mg/mL. These samples were incubated for 2 h at 37 °C and 150 rpm. To simulate intestinal conditions, the pH was increased to 7.0 with 1 M NaOH after addition of 7.7 mL simulated intestinal fluid (SIF), 28 μL CaCl_2_ and 917 μL double deionized water, and then 5.25 mL pancreatin and bile salt mixture were added at a final concentration of 0.175 and 1.10 mg/mL, respectively. The samples were incubated for 2 h at 37 °C and 150 rpm.

After gastric and intestinal digestion phases, samples were immediately cooled by immersion in an ice bath and then centrifuged at 10,000 rpm at 4 °C for 30 min (Megafuge 8R; Thermo Scientific, Darmstadt, Germany) to separate the bioaccessible fraction (BF) and the residual fraction. Bioaccessible fraction of the samples was used in further analysis. A blank sample (without the added sample) was also used to simulate gastrointestinal conditions and exclude interferences due to enzymes and digestion fluids.

Bioaccessibility was determined using equation (1) and expressed as percentage.

(1) $$\text{Bioaccessibility}\;\left(\%\right)=\left(\frac{{\text{BC}}_{\text{digested}}}{{\text{BC}}_{\text{non}-\text{digested}}}\right)x\;100$$

where BC_digested_ is the amount of bioactive compounds (TPC, DPPH, ABTS) obtained in the supernatants of the final digested sample solution (bioaccessible fraction) and BC_non-digested_ is the amount of bioactive compounds in non-digested sample solutions.

### 2.4 Determination of total phenolic content (TPC)

Total phenolic content were evaluated using the Folin-Ciocalteau reagent (Prior et al., 2005). Briefly, 20 μL of the sample or standard solution was mixed with 100 μL of the Folin-Ciocalteu reagent and left for incubation (5 min). Then, 100 μL of sodium carbonate solution (75 g/L in water) was added to the mixture. After incubation for 60 min at room temperature, absorbance values were recorded at 765 nm using an Infinite M200 spectrophotometer (Tecan, Crailsheim, Germany). Results were calculated according to the gallic acid calibration curve (R^2^ = 0.999) within the concentration range of 2.5–100 μg/mL and indicated as gram gallic acid equivalents (GAE) per L (g GAE/L). Water was used as blank.

### 2.5 Determination of the radical scavenging capacity

Antioxidant capacities of the samples were determined by applying ABTS (Re et al., 1999) and DPPH (Molyneux, 2004) radical scavenging ability assays. Trolox was used as a standard in both assays.

For ABTS radical scavenging ability, an ABTS radical stock solution was prepared by mixing ABTS (final concentration of 7 mM) with K_2_S_2_O_8_ (final concentration 2.5 mM) using double deionized water. The stock solution was stored at 4 °C for at least 16 h and protected from light. For ABTS working solutions, the stock solution was diluted with ethanol until an absorbance of 0.7 at 734 nm. Briefly, 10 μL of the sample or Trolox standard was mixed with 200 μL of the ABTS working solution. After incubation for 6 min at room temperature, absorbance values were recorded at 734 nm using an Infinite M200 spectrophotometer (Tecan). Results were calculated according to the Trolox calibration curve (R^2^ = 0.998) within the concentration range of 0.1–1.0 mmol/L and indicated as millimoles of Trolox equivalents (TE) per L (mmol TE/L).

For DPPH radical scavenging ability, DPPH stock solution (100 μM in ethanol) was diluted with ethanol to obtain a working solution of 30 μM, which was stored protected from light at 4 °C until further usage. Briefly, 100 μL of the sample or Trolox standard was mixed with 100 μL of the DPPH working solution. After incubation for 25 min at room temperature, absorbance values were recorded at 515 nm using an Infinite M200 spectrophotometer (Tecan). Results were calculated according to the Trolox calibration curve (R^2^ = 0.999) within the concentration range of 5–160 μmoL/L and indicated as μmoL TE per L (μmol TE/L). Ethanol was used as blank.

### 2.6 Cell culture study

For cell culture studies, human colon adenocarcinoma cells (Caco-2 cells) from the German Collection of Microorganisms and Cell Cultures (DSMZ; Braunschweig, Germany) were used. Also used were human colon cancer cells (HT29-MTX cells) from the European Collection of Authenticated Cell Cultures (ECACC; Porton Down, UK). The Caco-2 cells and HT29-MTX cells were cultured in Dulbecco's Modified Eagle's Medium (DMEM) containing 4.5 g/L glucose and stable glutamine supplemented with 10% (*v/v*) fetal bovine serum (FBS), 1% non-essential amino acids, 100 U/mL penicillin and 100 μg/mL streptomycin (all from Pan-Biotech, Aidenbach, Germany). The incubation of the two cell lines was carried out at 37 °C in a humidified atmosphere with 5% CO_2_. The medium was changed every 2–3 days. Trypsin-EDTA (0.05%) was used to passage the cells. The cells were passaged at a confluence of 70–80%. Cells with a passage number of 10–26 were used for the experiments.

To investigate transepithelial transport, Caco-2 and HT29-MTX cells were seeded together in inserts (0.4 μm pore diameter, 1x10^8^ pore density, 0.3 cm^2^ growth area insert, Sarstedt, Nümbrecht, Germany) of 24-well plates. For each experiment, 200 μL of growth medium with a density of 5 × 10^4^ cells/well was added to the apical compartment and 800 μL of growth medium was added to the basolateral compartment of the wells of a 24-well plate. Specifically, 3.5 × 10^4^ cells/well of Caco-2 cells and 1.5 × 10^4^ cells/well of HT29-MTX cells were seeded into the inserts. Prior to the transport experiments, the cells were differentiated for 21 days and formed converging monolayers after seeding. During differentiation over 21 days, the quality of the forming monolayer of Caco-2 and HT29-MTX cells was checked by measuring the transepithelial electrical resistance (TEER). Only differentiated cells with consistent TEER values were used for the transport experiment. The growth medium was replaced with fresh medium every two days during differentiation.

### 2.7 Cytotoxicity assay

The viability of the cells was determined using the resazurin-based assay, which quantifies the metabolic conversion of resazurin into the fluorescent compound resorufin (O'Brien et al., 2000). For this purpose, a total of 2.5 × 10^3^ cells were seeded in 96-well plates and differentiated into converging monolayers for 14 days after seeding. After 14 days, the cells were treated with the undigested or digested samples at dilution ratios of 1:10, 1:20 and 1:50 (*v/v*). In this case, Triton-X (1% (*v/v*)) was used as a positive control. After treating the cells with the samples for 24 h, the supernatant was rejected and the cells were incubated for 2 h with a resazurin solution diluted in the culture medium (10% *(v/v*); 100 μL/well). Afterwards, fluorescence was measured at 560 nm excitation and 690 nm emission in comparison to blank wells containing only medium. Analysis was performed spectrophotometrically using an Infinite M200 spectrophotometer (Tecan). This assay was performed in three biological replicates with three technical replicates per treatment.

Cell viability was calculated using the following equation (2):

(2) (mean fluorescence intensity of the treatment/mean fluorescence intensity of the control) × 100

The viability of the control was considered as 100%. Non-cytotoxic concentration of the test compounds was used for the following transport experiments.

### 2.8 Transport experiment

The transport experiment was performed according to the previously described procedure (Wu et al., 2017). Briefly, the growth medium in the apical and basolateral sides of the transwells was replaced with HBSS. After pre-incubation for 1 h, HBSS was removed. The sample solution (BF) used for the transport experiments was diluted at 1:20 (*v/v*) with HBSS, because, according to the results of the resazurin assay, the viability of the cells was more than 85–90% after 4 h of exposure to the samples with this concentration. The transport experiments were performed at 37 °C with 5% CO_2_ in air for 4 h. During transport, 100 μL of the samples were collected from both the apical and basolateral sides after 2 h and 4 h, respectively, and replaced with 100 μL of HBSS. The electrical resistance (TEER) was measured using the Millicell® ERS-2 unit equipped with an STX1 electrode (Millipore, Bedford, MA, USA) to assess the barrier function of the co-cultures. Cell culture inserts without cells were measured as reference. Cells with TEER value higher than 300 Ω were regarded as simulated intestinal epithelium and used in the transport experiment. Indeed, in the literature, TEER values of at least 200 Ωcm^2^ were recommended (Palm et al., 1996). Capric acid (C10, 10 mmol/L) was used as a positive control leading to a decrease in TEER values by enhance the tight junction permeability. The TEER value of the cells was monitored over a 24 h period (before, during and after treatment) of the experiments to maintain the integrity of the monolayer. After transport experiments, apical and basolateral compartments were filled with growth medium. The cells were then incubated again for 20 h to detect irreversible damage in the cell monolayer. All samples were stored at −80 °C until further analysis.

### *2.9* *In vitro*-permeability-assay with *Lucifer Yellow*

To determine the permeability of the intestinal barrier, the transport of *Lucifer Yellow* was determined. In brief, a *Lucifer Yellow* stock solution (450 μmol/L in HBSS) was diluted with HBSS to obtain a working solution of 3 μM *Lucifer Yellow*, which was stored protected from light at −20 °C until further use. Subsequently, 10 μL of the *Lucifer Yellow* stock solution (450 μmol/L in HBSS) was added to the apical chamber together with the sample solutions. After 4 h of incubation at 37 °C and 5 % CO_2_, *Lucifer Yellow* was detected in the apical and basolateral samples (100 μL) using a microplate reader (excitation λ = 428 nm, emission λ = 536 nm). *Lucifer Yellow* standard in the range of 0.05–0.45 μmol/L in HBSS was used for calibration. Results were expressed as the percentage of *Lucifer Yellow* substance in the apical compartment relative to the initial value of the simulated intestinal barrier.

### 2.10 Calculation of the apparent permeability coefficient (Papp)

The permeability coefficient (Papp) was calculated according to the following equation (3), (4):

(3) dQ=dCxV

where *dC* is the rate of change in concentration of test compound in the basal side and *V* is the volume of the receiver side:

(4) $$Papp=\frac{dQ}{dt}\;\frac{1}{AC_{0}}$$

*A* is the surface area of the polycarbonate membrane of the insert (cm^2^); *C*_*0*_ is the initial concentration of the test compound at the apical side (mM) and dt is the flux with Q, the amount of molecules transported over time (t) of the incubation.

### 2.11 Statistical analysis

Statistical analysis was carried out using the software Prism (version 10.1.1; GraphPad, La Jolla, CA, USA). Data were analyzed for normality of distribution by Shapiro–Wilk test, followed by a one-way ANOVA including Tukey's multiple comparison test for normally distributed data. Differences were considered as significant when *p* < 0.05, while data are shown as means ± SD (Standard Deviation) of at least three independent experiments.

## 3 Results

### 3.1 Change in the total phenolic content and antioxidant capacity of chokeberry beverages before *in vitro* digestion

**Fig. 1:**
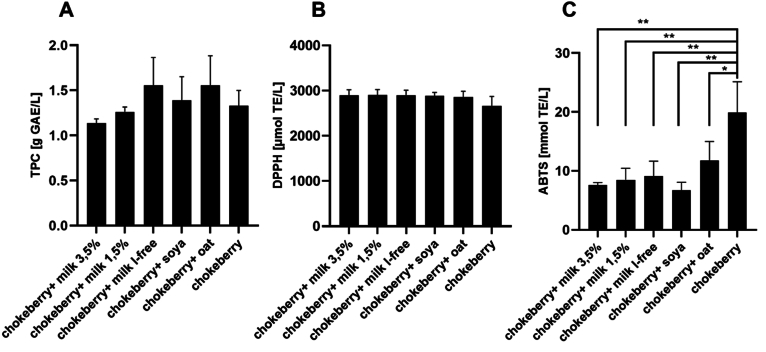
Total phenolic content and free radical scavenging activity of chokeberry juicebased beverages before *in vitro* digestion. **A**: Total phenolic content of the fruit juice extracts, GAE: Gallic acid equivalents; mean value ± standard deviation (n = 3); **B**: Antioxidant capacity of chokeberry juice-based beverages determined by the DPPH test. TE: Trolox equivalent; mean ± standard deviation (n = 3); **C**: Antioxidant capacity of chokeberry juice-based beverages determined by the ABTS assay. TE: Trolox equivalents; mean value ± standard deviation (n = 3); ∗*p* < 0.05, ∗∗*p* < 0.01 compared to chokeberry.

Fig. 1A shows the total phenolic content of chokeberry drinks prepared with different food matrices before intestinal digestion. Undigested, the sample solutions with chokeberry juice in combination with lactose-free milk and in combination with oat drink showed the highest total phenol content (1.55 g GAE/L). Chokeberry juice in combination with milk with a fat content of 3.5% had the lowest total phenolic content of 1.14 g GAE/L sample solution. However, there were no significant differences between the samples.

The antioxidant activity of the sample solutions is shown in Fig. 1B and C. Basically, Fig. 1B shows that the antioxidant capacity of the undigested sample solutions determined with the DPPH assay is similarly high and lies between 26575 and 2902 μmol TE/L sample solution.

In addition, the antioxidant capacity of the samples was determined by applying ABTS. Fig. 1C shows that the chokeberry juice alone before intestinal digestion with 19.90 mmol TE/L sample solution has the significantly highest ABTS radical scavenging property compared to all other sample solutions (*p* < 0.05). The sample solutions from chokeberry juice in combination with soy and cow's milk with 3.5% fat showed the lowest antioxidant activity.

### 3.2 Change in the total phenolic content and antioxidant capacity of chokeberry beverages after *in vitro* digestion

**Fig. 2:**
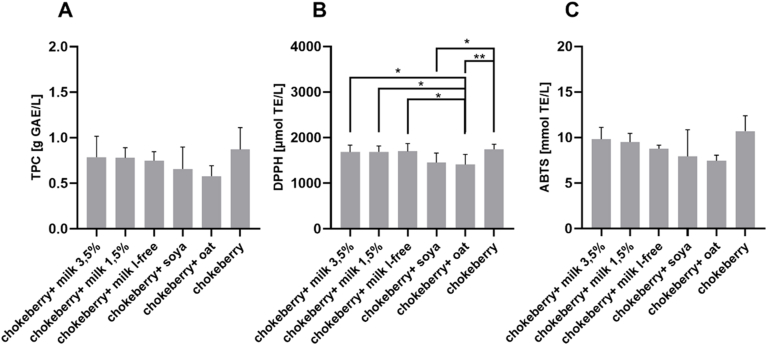
Total phenolic content and free radical scavenging activity of chokeberry juicebased beverages after *in vitro* digestion. **A**: Total phenolic content of the fruit juice samples, GAE: Gallic acid equivalents; mean value ± standard deviation (n = 3); **B**: Antioxidant capacity of chokeberry juice-based beverages determined by the DPPH test. TE: Trolox equivalents; mean ± standard deviation (n = 3); ∗*p* < 0.05, ∗∗*p* < 0.01; **C**: Antioxidant capacity of chokeberry juice-based beverages determined by the ABTS assay. TE: Trolox equivalents; mean value ± standard deviation (n = 3).

Fig. 2A shows the total phenolic content in chokeberry drinks after intestinal digestion. Chokeberry juice had the highest TPC with 0.87 g GAE/L sample solution. Chokeberry juice in combination with oat drink showed the lowest TPC after intestinal digestion (0.58 g GAE/L). Fig. 2B shows that the sample solution consisting of chokeberry juice has a significantly higher radical scavenging activity compared to the combination of chokeberry juice with soy or oat drink (*p* < 0.05). The sample solution consisting of chokeberry juice and oat drink has significantly lower radical scavenging activity compared to the sample solutions consisting of chokeberry juice in combination with cow's milk (*p* < 0.05).

The antioxidant capacity of the samples after intestinal digestion using ABTS is similarly high. The sample solution with chokeberry juice alone had the highest antioxidant capacity with 10.70 mmol TE/L. The sample solution consisting of chokeberry juice in combination with oat drink had the lowest antioxidant capacity with 7.50 mmol TE/L sample solution. These results are comparable with the antioxidant capacity determined by the DPPH assay. However, no significant differences between the sample solutions are recognizable here either (Fig. 2C).

**Fig. 3:**
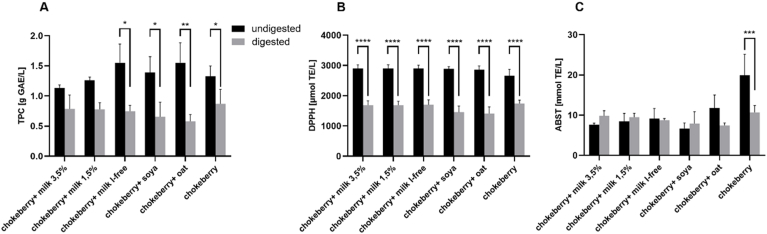
Total phenolic content and free radical scavenging activity of chokeberry juice-based beverages before and after *in vitro* digestion. **A**: TPC of the fruit juice extracts, GAE: Gallic acid equivalents; mean value ± standard deviation (n = 3); ∗*p* < 0.05, ∗∗*p* < 0.01 compared to the undigested sample solution. **B**: Antioxidant capacity of chokeberry juice-based beverages determined by the DPPH test. TE: Trolox equivalents; mean value ± standard deviation (n = 3); ∗∗∗∗*p* < 0.0001 compared to the undigested sample solution. **C**: Antioxidant capacity of chokeberry juice-based beverages determined by the ABTS test. TE: Trolox equivalents; mean value ± standard deviation (n = 3); ∗∗∗*p* < 0.001 compared to the undigested sample solution.

Overall, it is noticeable that the TPC content of the samples before simulated *in vitro* digestion was higher than after digestion. In addition, a significant decrease in TPC after intestinal digestion can be observed in the sample solutions with chokeberry juice in combination with lactose-free cow's milk, as well as with soy and oat drink and with chokeberry juice alone (*p* < 0.05). In the sample solutions consisting of chokeberry juice in combination with cow's milk with 3.5% and 1.5% fat, no significant differences in the total phenol content were observed before and after the simulated *in vitro* digestion (Fig. 3A).

Fig. 3B is shown that the radical scavenging activity of all samples decreases significantly after the simulated in-vitro digestion compared to the undigested samples. Fig. 3C shows that the radical scavenging activity determined by ABTS does not change significantly in any of the sample solutions after the simulated *in vitro* digestion compared to the undigested sample solutions. One exception, however, is the pure chokeberry juice, which at 19.90 mmol TE/L sample solution had the significantly highest undigested radical scavenging activity (*p* < 0.05). After simulated *in vitro* digestion, a significant decrease in radical scavenging activity to 10.70 mmol TE/L sample solution was observed in the chokeberry juice (*p* < 0.05). For all other sample solutions tested, the radical scavenging activity after intestinal digestion was 7.4-9.80 mmol TE/L sample solution.

**Fig. 4:**
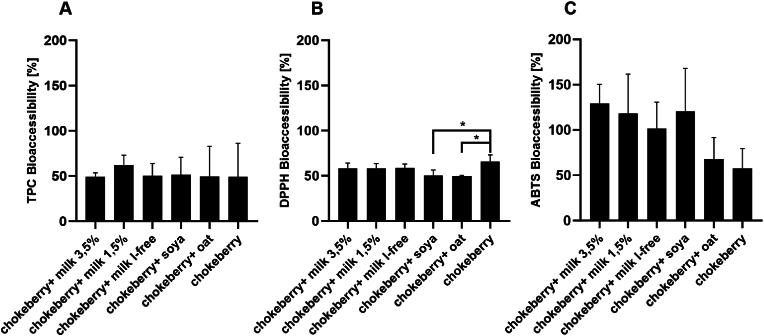
Bioaccessibility of bioactive compounds in chokeberry juice-based beverages. Results were shown as the mean and standard deviation of at least three independent passages; mean value ± standard deviation (n = 3), ∗*p* < 0.05 compared to chokeberry.

The effects of gastrointestinal digestion on the bioaccessibility of bioactive compounds in chokeberries in combination with milk and milk alternatives are shown in Fig. 4. The bioaccessibility of TPC was about 50% in all analyzed samples. The bioaccessibility of DPPH radical scavenging activity was 50%, with the exception of pure chokeberry juice, where it was 65% and thus significantly different from the bioaccessibility of chokeberry juice in combination with soy and oat drink (*p* < 0.05). Regarding the bioaccessibility of ABTS, TPC and DPPH, no significant differences were found between the individual sample solutions.

### 3.3 Effects of different food matrices on the transepithelial transportation of chokeberry juice bioactives

#### 3.3.1 Transepithelial electrical resistance (TEER)

**Fig. 5:**
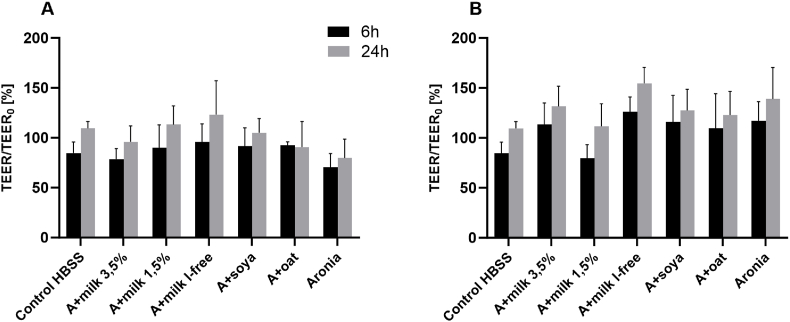
Change (%) in transepithelial electrical resistance values normalized to their initial (0 h) values. (A) undigested, treated with the undigested samples at 1:20 (*v/v*) dilution ratio. (B) digested, treated with the digested samples at 1:20 (*v/v*) dilution ratio, mean value ± standard deviation (n = 3).

To observe the permeability of the intestinal barrier during the treatment of Caco-2 and HT29-MTX cells, TEER values were measured during the treatment of cells. Furthermore, the TEER values were measured after the 4-h treatment over a period of up to 24 h after the start of treatment in order to observe any regeneration of the intestinal barrier. The TEER values determined at 6 and 24 h after the start of treatment were then compared with each other. The lowest TEER values were obtained at 6 h after the start of treatment, whereas the TEER values recovered 24 h after the start of treatment and approximately returned to the initial TEER value. As can be seen in Fig. 5A and B, the TEER value decreased 6 h after the start point of treatment compared to the initial value. The strongest decrease in TEER values to 70% compared to the initial TEER value was observed when the cells were treated with undigested chokeberry juice. There was no difference in the TEER values between undigested and digested samples 6 h after the start point of treatment compared to the control. At 24 h after the start of cell treatment, the TEER values increased significantly again compared to the TEER values after 6 h, and almost reached 100 % again compared to the initial TEER value. Thus, the TEER values stabilized again 24 h after the start of treatment and partially returned to the initial value at the start of treatment. It is also remarkable that the TEER values did not decrease as much when the cells were treated with the digested sample solution 6 h after the start of treatment as when they were treated with the undigested sample solutions. The cells also reached a TEER value 24 h after the start of treatment when treated with the digested sample solution, which was above 100% compared to the initial TEER value. It can therefore be assumed that the cells regenerated better 24 h after the start of treatment when treated with digested sample solutions.

#### 3.3.2 Permeability of *Lucifer Yellow*

**Fig. 6:**
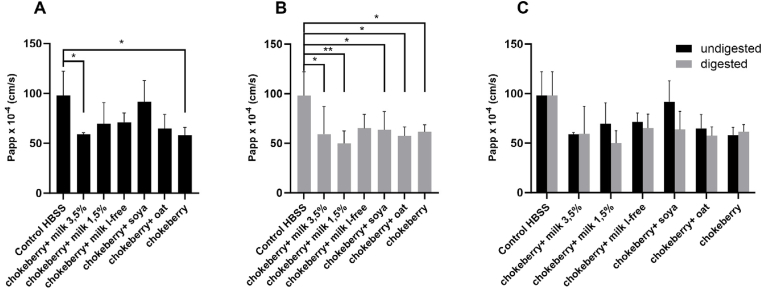
Effects of different food matrices on the permeability determined by *Lucifer Yellow*. (A) undigested, treated with the undigested samples at 1:20 (*v/v*) dilution ratio. (B) digested, treated with the digested samples at 1:20 (*v/v*) dilution ratio. (C) undigested and digested, treated with the undigested and digested samples at 1:20 (*v/v*) dilution ratio, mean value ± standard deviation (n = 3), ∗*p* < 0.05, ∗∗*p* < 0.01 compared to control.

To investigate whether the treatment with undigested or digested sample solutions consisting of chokeberry juice in combination with milks or milk alternatives have an influence on the permeability of the intestinal barrier, the paracellular flow test with *Lucifer Yellow* was carried out in the Caco-2/HT29-MTX co-culture model (Fig. 6).

As shown in Fig. 6A, the cells treated with undigested chokeberry juice in combination with soy drink have the highest Papp index or the highest flux of sodium fluorescein from the apical to the basolateral compartment compared to the other treatments after 4 h. The significantly lowest Papp index compared to the control (HBSS) was observed when cells were treated with chokeberry juice and chokeberry juice in combination with milk with a fat content of 3.5% (*p* < 0.05). All other treatment solutions show no significant influence on the transport of *Lucifer Yellow* through the intestinal membrane compared to the control. When the cells were treated with the digested sample solutions, the lowest Papp index was observed in the treatment with the digested chokeberry juice in combination with milk (1.5% fat). The Papp index can be seen in Fig. 6B and amounted to 50% after 4 h of the treatment and thus differed significantly from the Papp index of the control (*p* < 0.05). Basically, all Papp indices differed significantly from the control, with the exception of the Papp index when the cells were treated with lactose-free cow's milk (*p* < 0.05).

## 4 Discussion

To evaluate the influence of the food matrix on the total antioxidant capacity, the DPPH assay was applied for the digested and undigested samples. The sample solutions prepared in the current study, consisting of chokeberry juice, showed the highest antioxidant effect in the ABTS assay undigested with 5.0 g TE/L sample solution compared to the other sample solutions. According to Nowak et al. (2016), an antioxidant capacity of 4.9 mg TE per 100 g was determined for pure chokeberry juice (Nowak et al., 2016). The high antioxidant effect of chokeberry alone, as well as in combination with milk and milk alternatives, which was determined using the DPPH assay, was confirmed by the TPC assay. It can therefore be assumed that these sample solutions also have an almost equal antioxidant effect, which was confirmed by the DPPH assay. However, using the ABTS assay, a significantly lower antioxidant effect was observed in all sample solutions compared to chokeberry juice alone. It can therefore be assumed that chokeberry juice alone has one of the highest antioxidant effects of the sample solutions examined, even if the results of the ABTS and DPPH tests were different. While ABTS radicals are more accessible for electron transfer, the principle of the DPPH assay is based on hydrogen transfer (Wołosiak et al., 2021). Therefore, the measured mechanisms are not chemically identical and depend on the specific compounds. It has already been shown that polyphenols lead to different results in the antioxidant assays, which is why the use of more than one assay is recommended (Wołosiak et al., 2021; Csepregi et al., 2016). The decrease in the antioxidant capacity of undigested milk or milk alternative mixtures in the ABTS assay can also be attributed to the precipitation of proanthocyanidins by milk proteins. These reductions may be due to the complex formation between chokeberry polyphenols and milk proteins. Procyanidins can bind tightly to proline-rich proteins such as milk caseins at pH values close to or below the isoelectric point of the protein (Hagerman and Butler, 1980). This affinity also depends on the molecular weight, three-dimensional structure and proline content of the protein as well as the degree of polymerization of the procyanidins (Hagerman and Butler, 1980). The procyanidin/protein ratio is an important factor that determines the solubility, which, among other things, determines the complexes formed. While a high procyanidin/protein ratio promotes the formation of insoluble complexes, a lower procyanidin/protein ratio leads to the formation of soluble complexes (Adamczyk et al., 2012). After gastrointestinal digestion, a significant decrease in apparent DPPH values was observed in all sample solutions. The same results were observed with the TPC results, with the exception of the sample solutions consisting of chokeberry juice in combination with cow's milk with 3.5% and 1.5% fat content. In contrast, significant differences were observed in the antioxidant activity determined by the DPPH assay between the tested sample solutions. For example, the antioxidant activity of the sample solutions consisting of chokeberry juice and milk components differed significantly from the sample solution consisting of chokeberry juice in combination with oat drink.

An average bioaccessibility of 40–60% was achieved for all samples. These results are comparable with the results of Buniowska et al. (2017). In this study, the bioaccessibility of TPC in a fruit juice mixture of mango and papaya was determined to be 30.5%. In addition, another study (Rodríguez-Roque et al., 2015) found a slightly lower bioaccessibility of TPC in a fruit juice mixture of orange, kiwi, pineapple and mango at 25.9% and 15.1%, respectively (Rodríguez-Roque et al., 2015). Furthermore, according to Rodríguez-Roque et al. (2014), fruit polyphenols from fruits are absorbed under digestive conditions through polymerization, epimerization and autooxidation (Rodríguez-Roque et al., 2014). Due to their low stability under alkaline conditions, degradation of phenolic substances is possible throughout gastrointestinal digestion. This finding is consistent with the changes observed in this study in the TPC and DPPH assays after *in vitro* digestion. Although the release of bioactives from the food matrix can be accelerated under the influence of digestive enzymes, temperature and pH conditions of intestinal digestion, it has also been reported that the bioaccessibility of anthocyanins is generally lower than that of other flavonoids (Yang et al., 2011). According to Kamiloglu et al. (2017), the high loss of anthocyanins could be due to a possible structural rearrangement due to pH changes in the molecular structures (Kamiloglu et al., 2017).

Ozkan et al. (2022) reported that the important physiological functions and prebiotic effects are due to a large number of soluble dietary fibers (e.g. pectin) found in raw fruits (Ozkan et al., 2022). However, molecular interactions between the polyphenols and pectin can impair the release of the polyphenols from the food matrix. Numerous studies have already found a negative influence of dietary fiber on the bioaccessibility of polyphenols. Indeed, due to the fact that oat and soy drink-based chokeberry beverages contain higher dietary fiber content, it was found that there was a declining tendency in the TPC and antioxidant capacity of the beverages prepared with oat or soy drinks after digestion. Similarly, the joint digestion of pectin and pomegranate led to a 2-fold decrease in the TPC value of the digested fraction. The possibly resulting fiber-polyphenol complex can hinder the release and thus the absorption of polyphenols in the intestine; this complex formation thus has a negative effect on *in vitro* bioaccessibility.

Based on these previous studies, it can be hypothesized that the protective effect of milk and milk alternatives on the polyphenols in chokeberry during gastrointestinal digestion via protein-phenol interactions lead to a reduced digestive loss of polyphenols, improved bioaccessibility and thus higher bioactivity.

In addition to these, it is still unclear to what extent the fat content of the cow's milk could play a relevant role. With regard to the different fat content of the cow's milk samples used in this study, no significant differences can be identified in terms of total phenolic content or antioxidant activity. According to Quan et al. (Quan et al., 2020; Rodríguez-Roque et al., 2015), the fat content of milk favors the uptake of bioactive compounds (especially lipophilic compounds) in micelles, which may lead to a higher bioactive amount. Since most polyphenols are water-soluble and transported in the body directly via the portal vein, it is assumed that dietary fats may have a limited effect on the more hydrophilic polyphenols. The situation may be different for the more hydrophobic components. Dietary fats have also been shown to increase gastrointestinal transit and could therefore alter the kinetics of polyphenol absorption. When strawberries with cream were consumed in a human test, a delayed but not altered total bioaccessibility of anthocyanins in plasma was observed over a 24-h period (Mullen et al., 2008). Furthermore, studies by Langley-Evans et al. (Langley-Evans, 2000) showed that high-fat milk digested *in vitro* with black tea had a significantly stronger negative effect on antioxidant capacity than low-fat milk (Langley-Evans, 2000). According to Serafini et al. (2009), one reason for this could be that longer peptide chains, the increase of which is apparently associated with increasing fat content, could have led to increased complexation of the polyphenols (Serafini et al., 2009). In contrast, the presence of lactose does not appear to have any effect on intestinal permeability or on the transport of *Lucifer Yellow* through the intestinal membrane compared to the lactose-free sample solutions. Therefore, it is assumed that lactose does not seem to have any influence on intestinal permeability.

Another aim of this study was to evaluate the effects of milk and milk alternatives on the bioavailability of chokeberry polyphenols. It can be assumed that a decrease in TEER values indicates an increase in intestinal permeability. After treatment of the cells with digested and undigested chokeberry juice in combination with milk and milk alternatives, a slight decrease in TEER values was observed as early as 6 h after the start of treatment when the cells were treated with the undigested sample solutions. However, 24 h after the start of treatment, almost complete regeneration of intestinal permeability was observed when treated with the digested and undigested sample solutions.

It can therefore be concluded that the influence of the different sample solutions is not necessarily due to their composition in relation to the milk and milk alternatives, but perhaps rather to the changes in the matrix that occurred during *in vitro* digestion. The flow rate of the transport marker *Lucifer yellow* and the Papp index calculated from it show that the index tends to have higher values when the cells are treated with the undigested sample solutions than with the digested sample solutions. This confirms the changes in the TEER values, as higher TEER values were also achieved in the treatment with the digested sample solutions than with the undigested sample solutions, which allows the conclusion that in this case there is a lower intestinal permeability, so that more fluorescent dye can be transported through the intestinal membrane. However, it should be noted that substances cannot only be transported through the intestinal membrane by increasing permeability, but that transport can also occur via other intracellular transport mechanisms. This could mean that transport of *Lucifer Yellow* could also occur intracellularly, which would then remain unobserved if permeability alone were determined by the TEER values. As already mentioned, the significance of TEER measurements is sometimes severely limited by external influencing factors such as temperature. In addition, TEER values depend on paracellular ion fluxes such as sodium and chloride (Suzuki and Hara, 2009). For example, Xie and coworkers (Xie et al., 2013) describe somewhat different observations to the results presented in our study (Xie et al., 2013). In the study by Xie et al. (2013), the effects of milk supplementation on the bioaccessibility of catechins found in green tea, which are also naturally found in larger quantities in chokeberry, and on intestinal absorption were investigated in an *in vitro* digestion/Caco-2 cell model (Xie et al., 2013). Catechins from digested green teawith 10 and 25% added milk showed increased intestinal permeability in the Caco-2 model compared to undigested green tea catechins and digested green tea catechins without adding milk (Xie et al., 2013).

In summary, the results clearly show that the presence of different food matrices containing protein, fat, sugar and minerals, as well as their content, have a major influence on the stability, bioaccessibility and intestinal absorption of phenolic compounds. In principle, pure chokeberry juice as well as chokeberry juice in combination with cow's milk shows a higher bioaccessibility in the DPPH assay than in combination with the milk alternatives soy and oats. However, there are no significant differences in the combination of chokeberry juice with milk in terms of lactose content and fat content.

## CRediT authorship contribution statement

**Magdalena Köpsel:** Investigation (chemical analyses, cell culture studies), Writing – original draft, Writing – review & editing, Visualization. **Gulay Ozkan:** Investigation (cell culture studies), Writing – original draft, Writing – review & editing, Visualization. **Tuba Esatbeyoglu:** Conceptzualization, Supervision, Project administration, Funding acquisition, Writing – review & editing.

## Funding

The publication of this article was funded by the Open Access Fund of Leibniz Universität Hannover.

## Declaration of competing interest

The authors declare that they have no known competing financial interests or personal relationships that could have appeared to influence the work reported in this paper.

## Data Availability

Data will be made available on request.

## References

[bib1] Adamczyk B., Salminen J.-P., Smolander A., Kitunen V. (2012). Precipitation of proteins by tannins: effects of concentration, protein/tannin ratio and pH. Int J of Food Sci Tech.

[bib2] Adolfsson O., Meydani S.N., Russell R.M. (2004). Yogurt and gut function. Am. J. Clin. Nutr..

[bib3] Anderson R.C., Cookson A.L., McNabb W.C., Park Z., McCann M.J., Kelly W.J., Roy N.C. (2010). Lactobacillus plantarum MB452 enhances the function of the intestinal barrier by increasing the expression levels of genes involved in tight junction formation. BMC Microbiol..

[bib4] Antunes F., Andrade F., Araújo F., Ferreira D., Sarmento B. (2013). Establishment of a triple co-culture *in vitro* cell models to study intestinal absorption of peptide drugs. Eur. J. Pharm. Biopharm..

[bib5] Astrup A. (2014). Yogurt and dairy product consumption to prevent cardiometabolic diseases: epidemiologic and experimental studies. Am. J. Clin. Nutr..

[bib6] Bermúdez-Soto M.J., Tomás-Barberán F.A., García-Conesa M.T. (2007). Stability of polyphenols in chokeberry (*Aronia melanocarpa*) subjected to *in vitro* gastric and pancreatic digestion. Food Chem..

[bib7] Brodkorb A., Egger L., Alminger M., Alvito P., Assunção R., Ballance S., Bohn T., Bourlieu-Lacanal C., Boutrou R., Carrière F., Clemente A., Corredig M., Dupont D., Dufour C., Edwards C., Golding M., Karakaya S., Kirkhus B., Le Feunteun S., Lesmes U., Macierzanka A., Mackie A.R., Martins C., Marze S., McClements D.J., Ménard O., Minekus M., Portmann R., Santos C.N., Souchon I., Singh R.P., Vegarud G.E., Wickham M.S.J., Weitschies W., Recio I. (2019). INFOGEST static *in vitro* simulation of gastrointestinal food digestion. Nat. Protoc..

[bib8] Buniowska M., Carbonell-Capella J.M., Frigola A., Esteve M.J. (2017). Bioaccessibility of bioactive compounds after non-thermal processing of an exotic fruit juice blend sweetened with *Stevia rebaudiana*. Food Chem..

[bib9] Calder P.C., Ahluwalia N., Brouns F., Buetler T., Clement K., Cunningham K., Esposito K., Jönsson L.S., Kolb H., Lansink M., Marcos A., Margioris A., Matusheski N., Nordmann H., O’Brien J., Pugliese G., Rizkalla S., Schalkwijk C., Tuomilehto J., Wärnberg J., Watzl B., Winklhofer-Roob B.M. (2011). Dietary factors and low-grade inflammation in relation to overweight and obesity. Br. J. Nutr..

[bib10] Cani P.D., Amar J., Iglesias M.A., Poggi M., Knauf C., Bastelica D., Neyrinck A.M., Fava F., Tuohy K.M., Chabo C., Waget A., Delmée E., Cousin B., Sulpice T., Chamontin B., Ferrières J., Tanti J.-F., Gibson G.R., Casteilla L., Delzenne N.M., Alessi M.C., Burcelin R. (2007). Metabolic endotoxemia initiates obesity and insulin resistance. Diabetes.

[bib11] Cao Y., Xiong Y.L. (2017). Interaction of whey proteins with phenolic derivatives under neutral and acidic pH conditions. J. Food Sci..

[bib12] Carrasco-Pozo C., Morales P., Gotteland M. (2013). Polyphenols protect the epithelial barrier function of Caco-2 cells exposed to indomethacin through the modulation of occludin and zonula occludens-1 expression. J. Agric. Food Chem..

[bib14] Costa J., Ahluwalia A. (2019). Advances and current challenges in intestinal *in vitro* model engineering: a digest. Front. Bioeng. Biotechnol..

[bib15] Csepregi K., Neugart S., Schreiner M., Hideg É. (2016). Comparative evaluation of total antioxidant capacities of plant polyphenols. Molecules.

[bib16] Cui W., Li L.X., Sun C.M., Wen Y., Zhou Y., Dong Y.L., Liu P. (2010). Tumor necrosis factor alpha increases epithelial barrier permeability by disrupting tight junctions in Caco-2 cells. Braz. J. Med. Biol. Res..

[bib19] Dima C., Assadpour E., Dima S., Jafari S.M. (2020). Bioavailability and bioaccessibility of food bioactive compounds; overview and assessment by *in vitro* methods. Compr. Rev. Food Sci. Food Saf..

[bib20] Dragovic-Uzelac V., Levaj B., Mrkic V., Bursac D., Boras M. (2007). The content of polyphenols and carotenoids in three apricot cultivars depending on stage of maturity and geographical region. Food Chem..

[bib21] Fresco P., Borges F., Diniz C., Marques M.P.M. (2006). New insights on the anticancer properties of dietary polyphenols. Med. Res. Rev..

[bib22] Gośliński M., Nowak D., Szwengiel A. (2021). Multidimensional comparative analysis of bioactive phenolic compounds of honeys of various origin. Antioxidants.

[bib23] Hagerman A.E., Butler L.G. (1980). Determination of protein in tannin-protein precipitates. J. Agric. Food Chem..

[bib24] Han X., Liang Z., Tian S., Liu L., Wang S. (2022). Epigallocatechin gallate (EGCG) modification of structural and functional properties of whey protein isolate. Food Res. Int..

[bib26] Kamiloglu S., Ozkan G., Isik H., Horoz O., van Camp J., Capanoglu E. (2017). Black carrot pomace as a source of polyphenols for enhancing the nutritional value of cake: an *in vitro* digestion study with a standardized static model. Food Sci. Technol..

[bib27] Kundu P., Dhankhar J., Sharma A. (2018). Development of non dairy milk alternative using soymilk and almond milk. Curr. Res. Nutr. Food Sci..

[bib28] Langley-Evans S.C. (2000). Antioxidant potential of green and black tea determined using the ferric reducing power (FRAP) assay. Int. J. Food Sci. Nutr..

[bib31] Lotito S.B., Frei B. (2006). Dietary flavonoids attenuate tumor necrosis factor alpha-induced adhesion molecule expression in human aortic endothelial cells. Structure-function relationships and activity after first pass metabolism. J. Biol. Chem..

[bib32] Manganaris G.A., Goulas V., Vicente A.R., Terry L.A. (2014). Berry antioxidants: small fruits providing large benefits. J. Sci. Food Agric..

[bib34] Meydani S.N., Ha W.K. (2000). Immunologic effects of yogurt. Am. J. Clin. Nutr..

[bib35] Molyneux P. (2004). The use of the stable free radical diphenylpicrylhydrazyl (DPPH) for estimating antioxidant activity. Songklanakarin J. Sci. Technol..

[bib36] Moreno-Navarrete J.M., Sabater M., Ortega F., Ricart W., Fernández-Real J.M. (2012). Circulating zonulin, a marker of intestinal permeability, is increased in association with obesity-associated insulin resistance. PLoS One.

[bib37] Mullen W., Edwards C.A., Serafini M., Crozier A. (2008). Bioavailability of pelargonidin-3-O-glucoside and its metabolites in humans following the ingestion of strawberries with and without cream. J. Agric. Food Chem..

[bib38] Nowak D., Gośliński M., Wojtowicz E. (2016). Comparative analysis of the antioxidant capacity of selected fruit juices and nectars: chokeberry juice as a rich source of polyphenols. Int. J. Food Prop..

[bib39] Nowak D., Gośliński M., Kłębukowska L. (2022). Antioxidant and antimicrobial properties of selected fruit juices. Plant Foods Hum. Nutr..

[bib40] Olas B. (2018). Berry phenolic antioxidants - implications for human health?. Front. Pharmacol..

[bib41] Ozkan G., Kostka T., Dräger G., Capanoglu E., Esatbeyoglu T. (2022). Bioaccessibility and transepithelial transportation of cranberrybush (*Viburnum opulus*) phenolics: effects of non-thermal processing and food matrix. Food Chem..

[bib42] O'Brien J., Wilson I., Orton T., Pognan F. (2000). Investigation of the alamar blue (resazurin) fluorescent dye for the assessment of mammalian cell cytotoxicity. Eur. J. Biochem..

[bib43] Pal S., Saha C., Hossain M., Dey S.K., Kumar G.S. (2012). Influence of galloyl moiety in interaction of epicatechin with bovine serum albumin: a spectroscopic and thermodynamic characterization. PLoS One.

[bib44] Palm K., Luthman K., Ungell A.L., Strandlund G., Artursson P. (1996). Correlation of drug absorption with molecular surface properties. J. Pharm. Sci..

[bib45] Prior R.L., Wu X., Schaich K. (2005). Standardized methods for the determination of antioxidant capacity and phenolics in foods and dietary supplements. J. Agric. Food Chem..

[bib46] Qin D., Yang X., Gao S., Yao J., McClements D.J. (2017). Influence of dietary fibers on lipid digestion: comparison of single-stage and multiple-stage gastrointestinal models. Food Hydrocolloids.

[bib47] Quan W., Tao Y., Qie X., Zeng M., Qin F., Chen J., He Z. (2020). Effects of high-pressure homogenization, thermal processing, and milk matrix on the *in vitro* bioaccessibility of phenolic compounds in pomelo and kiwi juices. J. Funct.Foods.

[bib48] Rafiee Z., Nejatian M., Daeihamed M., Jafari S.M. (2019). Application of different nanocarriers for encapsulation of curcumin. Crit. Rev. Food Sci. Nutr..

[bib49] Re R., Pellegrini N., Proteggente A., Pannala A., Yang M., Rice-Evans C. (1999). Antioxidant activity applying an improved ABTS radical cation decolorization assay. Free Radic. Biol. Med..

[bib51] Rodríguez-Roque M.J., Rojas-Graü M.A., Elez-Martínez P., Martín-Belloso O. (2014). *In vitro* bioaccessibility of health-related compounds from a blended fruit juice–soymilk beverage: influence of the food matrix. J. Funct. Foods.

[bib52] Rodríguez-Roque M.J., de Ancos B., Sánchez-Moreno C., Cano M.P., Elez-Martínez P., Martín-Belloso O. (2015). Impact of food matrix and processing on the *in vitro* bioaccessibility of vitamin C, phenolic compounds, and hydrophilic antioxidant activity from fruit juice-based beverages. J. Funct. Foods.

[bib53] Serafini M., Testa M.F., Villaño D., Pecorari M., van Wieren K., Azzini E., Brambilla A., Maiani G. (2009). Antioxidant activity of blueberry fruit is impaired by association with milk. Free Radic. Biol. Med..

[bib54] Shen F., Niu F., Li J., Su Y., Liu Y., Yang Y. (2014). Interactions between tea polyphenol and two kinds of typical egg white proteins—ovalbumin and lysozyme: effect on the gastrointestinal digestion of both proteins *in vitro*. Food Res. Int..

[bib55] Suzuki T., Hara H. (2009). Quercetin enhances intestinal barrier function through the assembly of zonula corrected occludens-2, occludin, and claudin-1 and the expression of claudin-4 in Caco-2 cells. J. Nutr..

[bib56] Swieca M., Sęczyk L., Gawlik-Dziki U., Dziki D. (2014). Bread enriched with quinoa leaves - the influence of protein-phenolics interactions on the nutritional and antioxidant quality. Food Chem..

[bib57] Treutter D. (2006). Significance of flavonoids in plant resistance: a review. Environ. Chem. Lett..

[bib58] Winuprasith T., Khomein P., Mitbumrung W., Suphantharika M., Nitithamyong A., McClements D.J. (2018). Encapsulation of vitamin D3 in pickering emulsions stabilized by nanofibrillated mangosteen cellulose: impact on *in vitro* digestion and bioaccessibility. Food Hydrocolloids.

[bib59] Wołosiak R., Drużyńska B., Derewiaka D., Piecyk M., Majewska E., Ciecierska M., Worobiej E., Pakosz P. (2021). Verification of the conditions for determination of antioxidant activity by ABTS and DPPH assays-A practical approach. Molecules.

[bib60] Wu T., Grootaert C., Voorspoels S., Jacobs G., Pitart J., Kamiloglu S., Possemiers S., Heinonen M., Kardum N., Glibetic M., Smagghe G., Raes K., van Camp J. (2017). Aronia (*Aronia melanocarpa*) phenolics bioavailability in a combined *in vitro* digestion/Caco-2 cell model is structure and colon region dependent. J. Funct. Foods.

[bib61] Xie Y., Kosińska A., Xu H., Andlauer W. (2013). Milk enhances intestinal absorption of green tea catechins in *in vitro* digestion/Caco-2 cells model. Food Res. Int..

[bib62] Yang M., I Koo S., O Song W., K Chun O. (2011). Food matrix affecting anthocyanin bioavailability. Curr. Med. Chem..

[bib63] Yildirim-Elikoglu S., Erdem Y.K. (2018). Interactions between milk proteins and polyphenols: binding mechanisms, related changes, and the future trends in the dairy industry. Food Rev. Int..

